# Effect of micrografting technique on growth and cold resistance of tea (*Camellia sinensis*) plant

**DOI:** 10.1186/s12870-025-06789-z

**Published:** 2025-06-02

**Authors:** Yaohua Cheng, Mengling Lin, Hongying Wang, Ziwen Zhou, Linxin Long, Qi Yang, Qiuyan Ban, Xianchen Zhang, Yeyun Li

**Affiliations:** 1 National Key Laboratory for Tea Plant Germplasm Innovation and Resource Utilization, Anhui Agricultural University, Hefei, 230036 China; 2 Minqing Meigu State-owned Forest Farm, Fuzhou, Fujian 350805 China; 3 College of Horticulture, Henan Agricultural University, Zhengzhou, 450000 China

**Keywords:** *Camellia sinensis*, Micrografting, Rootstock and scion selection, Cold tolerance

## Abstract

**Background:**

Micrografting technology has gained popularity in model plants, with the advantages of a wide grafting range and small space. However, this technique has not been fully explored in tea plants.

**Results:**

In our study, different rootstocks [radicle (obtained from the germination in seed), epicotyl without cotyledons, epicotyl with cotyledons, tea varieties] and scion (red branch, green branch) grafting combinations were used to estimate the survival rate, plant growth, the compatibility behavior, and cold tolerance of grafted seedlings. Our results showed that the higher survival rate and shooting rate were observed in radicle (obtained from the germinated seed diameter ≥ 15 mm, D3) as the rootstock. Also, the same growth indicators were found in the green branch as scion and radicle as rootstock (GB\R) were higher than that of other grafting combinations. In addition, the grafted seedlings of LJ43 as rootstock had the best growth rate, and the vascular bundle bridge was completely established in SCZ as scion and LJ43 as rootstock (SCZ/LJ43) graft combination, accompanied with a higher survival rate, shoot rate and leaf number of new shoots and cold tolerance in field experiments.

**Conclusion:**

Our findings provide a viable tea micrografting method, which has the potential to substitute traditional tea cuttings for tea seedling propagation and thus meet the requirements of tea cultivation.

## Background

Tea plant (*Camellia sinensis* (L.) O. Kuntze), an important economic woody crop [1], are mainly propagated by cutting [2]. With global weather patterns shifting towards worse trends, tea planting is seriously negatively affected by various abiotic stresses, particularly cold, drought and heat [3–5]. As is well known, plant grafting played an important role in enhancing their resistance to abiotic stresses, thus, the technique is widely used [6–8]. Tea grafting was initially used in the 1970s as a cost-effective means to improve low-yielding and inefficient tea plantations [9]. However, studies about grafting propagation in tea plants (*Camellia sinensis*) was limited.

Plant grafting propagation technology mainly includes woody grafting, vegetable grafting and micrografting techniques. Recently, micrografting (grafting with small, young tissues) has gained popularity for a wide range of grafted species and small space [10]. Micrografting techniques, including butt-end hypocotyl grafting, embryo grafting, and inflorescence grafting, was a highly efficient method in the breeding of *Camellia oleifera* [11], tobacco [12] and *Arabidopsis* [13]. In addition, the micrografting method, grafting time, rootstock, and scion variety. are important factors associated with the survival rate of micrografting [14]. Inflorescence micrografting-generated *Arabidopsis thaliana* grafting seedlings had an absolute advantage on the growth of scion [15]. Nowadays, better growth and higher survival of *Camellia oleifera* micrografting seedlings were found in suitable grafting time (16-20th May) [16]. Micrograft incompatibility (rootstock and scion selection) has been described in *Arabidopsis thaliana* grafting with cabbage (*Brassica*), radish (*Raphanus*), and tomato [15].

Recently, woody grafting (side-veneer grafting and cleft grafting) are the common grafting methods in tea plants [17, 18]. Although these methods are mature, they have certain limitations. Micro-grafting technology breaks through the traditional grafting’s dependence on seasons and temperatures, providing a more flexible and efficient means for plant propagation. However, studies about micrografting propagation in tea plants (*Camellia sinensis*) was limited. The main aims of the present study were (1) to identify the differences in the growth and survival of tea seedlings with different tea seed diameter and rootstock-scion combinations and (2) analyze the compatibility and cold resistance of different tea varieties as rootstock grafted seedlings to provide valuable information for the application of tea micrografting technology in the propagation of tea seedlings.

## Results and discussion

### Effect of the tea seed diameter on the growth of grafted seedlings

**Fig. 1:**
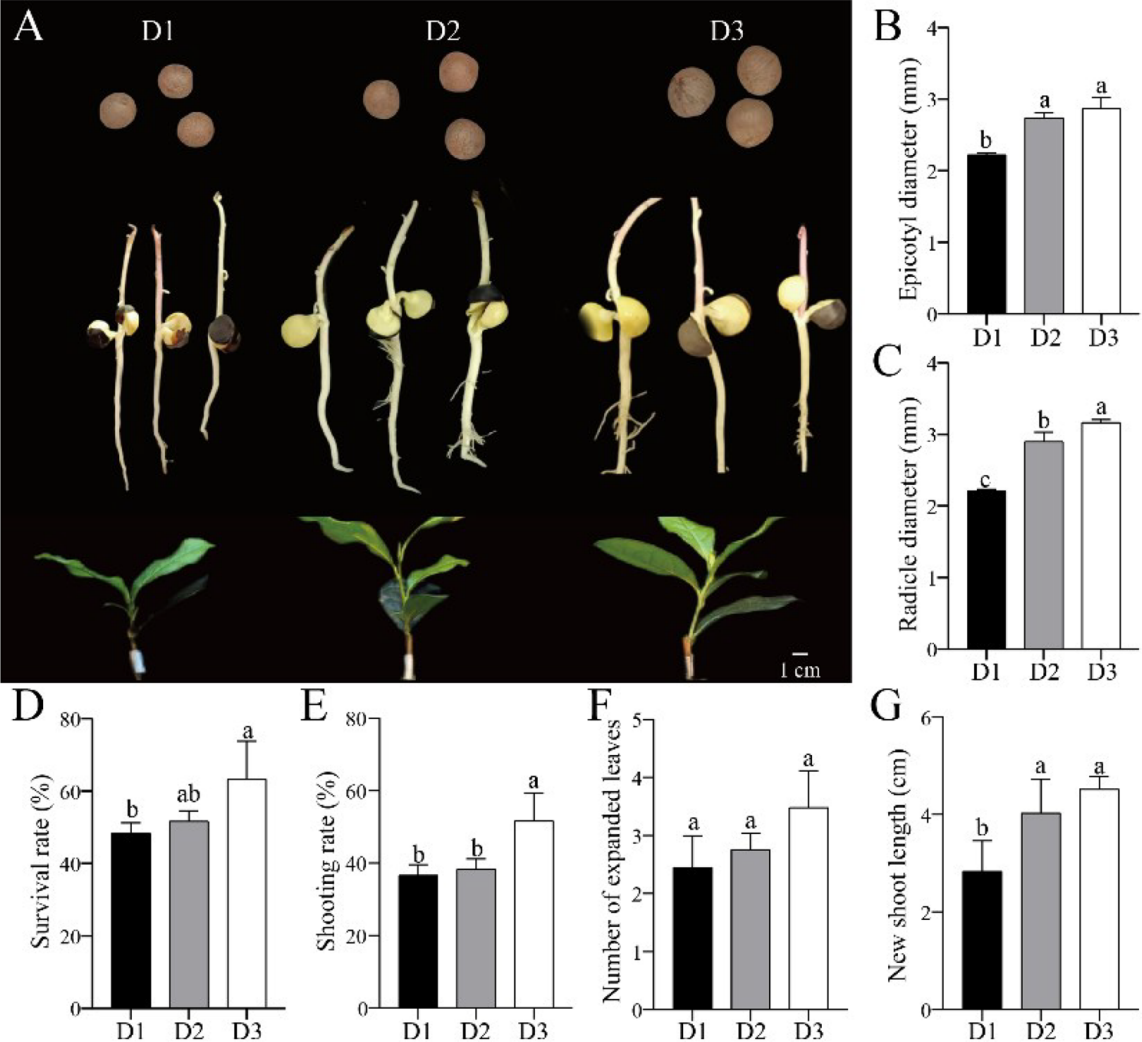
The growth status of tea seeds and the seedling phenotype for 120 d after grafting (**A**), the diameter of epicotyl (**B**), the diameter of radicle (**C**), the survival rate of grafted seedlings (**D**), the rate of shoot (**E**), the number of expanded leaves (**F**) and the length of new shoots (**G**). D1: 12 ≤ seed diameter<13.50 mm; D2: 13.50 ≤ seed diameter<15 mm; D3: seed diameter ≥ 15 mm. Different letters within each main factor indicate significance at Tukey’s HSD test (*P* ≤ 0.05)

Tea plants (*Camellia sinensis*) and oil tea plants (*Camellia oleifera*) belong to the genus *Camellia*, and the tea plants are genetically closely related to oil tea plants [19]. Based on the micro-grafting technology of oil tea plants, the germinated tea seeds were used to obtain radicles as rootstocks for grafting [11]. The seed diameter plays a critical role in determining the radicle diameter, which directly impacts the feasibility of grafting process, as well as the survival rate and subsequent growth performance of grafted seedlings. The rootstocks with better growth status exhibited a high level of division healing ability [20]. To explore the effect of radicle grown from different diameter of tea seeds on the growth of grafted seedlings, some experiments were conducted (Fig. 1A). In our study, D2 (13.50 ≤ seed diameter<15 mm) and D3 (seed diameter ≥ 15 mm) clearly exhibited a higher epicotyl diameter compared to D1 (12 ≤ seed diameter<13.50 mm) (Fig. 1B) and radicle diameter (Fig. 1C). The radicles of D2 and D3 groups were used as rootstocks, and the growth of grafted seedlings was much better than that of D1 (12 ≤ seed diameter<13.50 mm) (Fig. 1A). Consistent with the phenotype, the higher survival rate (Fig. 1D), shooting rate (Fig. 1E) and new shoot length (Fig. 1F) were observed in D3 group. A similar result reported that the selection of high-quality *Camellia oleifera* seeds with higher weights as rootstocks was beneficial for promoting the growth of grafted seedlings [21]. Therefore, D3 group was used for further experiments.

### Effects of different scion and rootstock on the growth of grafted tea seedlings

**Fig. 2:**
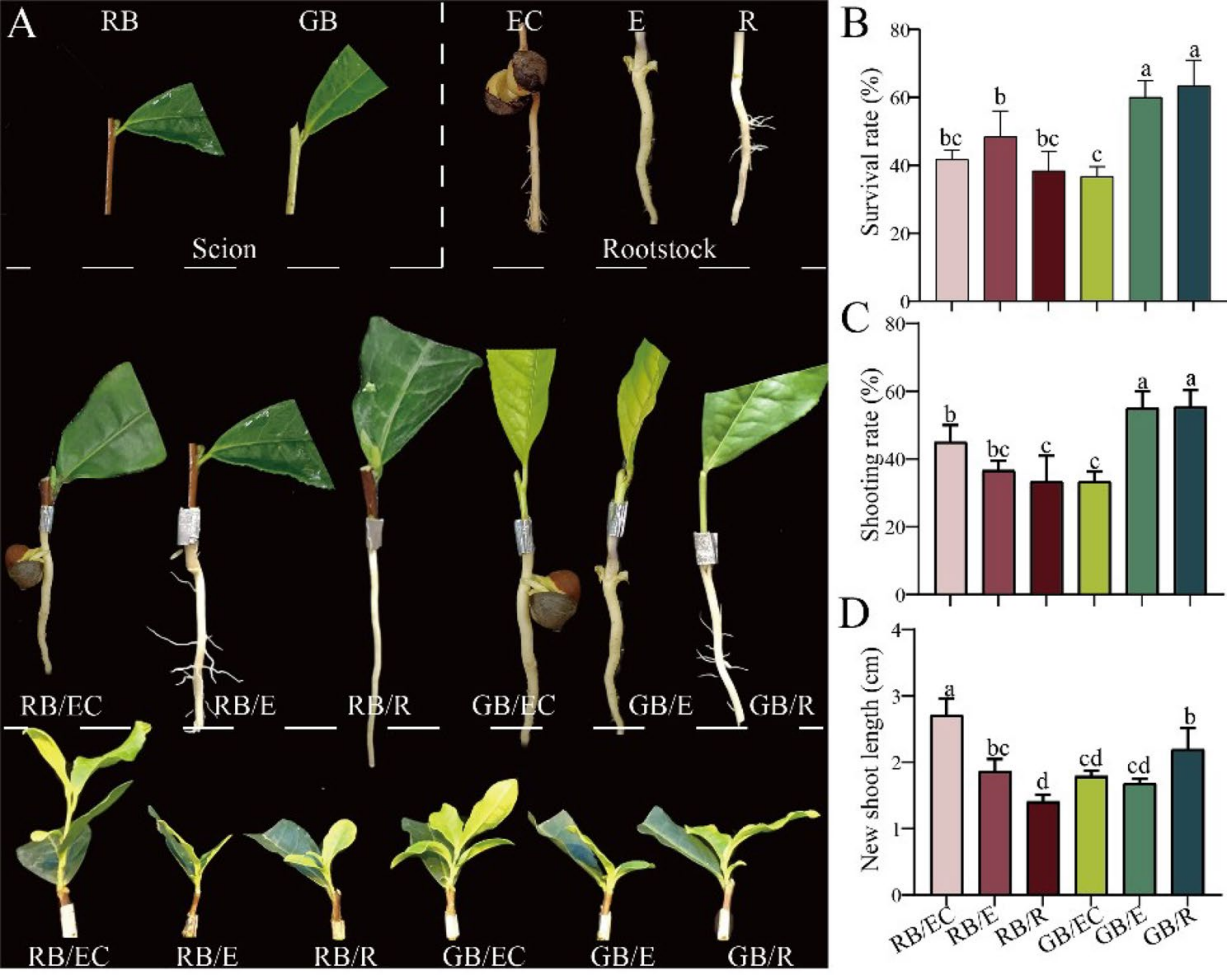
The growth status of grafted seedlings of different rootstocks and scion types (**A**), the survival rate of grafted seedlings (**D**), the rate of shoot (**C**) and the length of new shoots (**D**). RB: red branch; GB: green branch; EC: epicotyl with cotyledons; E: epicotyl without cotyledons; R: radicle

The degree of lignification of the scion and rootstock type also directly affects the survival rate of grafting [22, 23]. Therefore, the effect of scion [(GB) green branch and (RB) red branch] and rootstock [(EC) epicotyl with cotyledons, (E) epicotyl without cotyledons and (R) radicle] on the grafting growth of tea plants were studied as shown in Fig. 2A. In our study, the higher survival values (Fig. 2B) and higher shoot rate (Fig. 2C) were found in GB/E and GB/R. Also, the shoot length of GB/R was significantly higher than that of GB/E (Fig. 2D). Similarly, tobacco seed radicles as rootstocks can improve the reproductive growth and healing degree of grafted seedlings [12]. In addition, green branches as scions obtained higher survival rates in *Camellia oleifera* compared with the other scions group [24]. Consistent with the previous study, our results confirmed that the graft combination GB/R had a maximum positive effect on the growth of grafted tea seedlings.

### Effects of different rootstocks on compatibility of the grafted tea seedlings

**Fig. 3:**
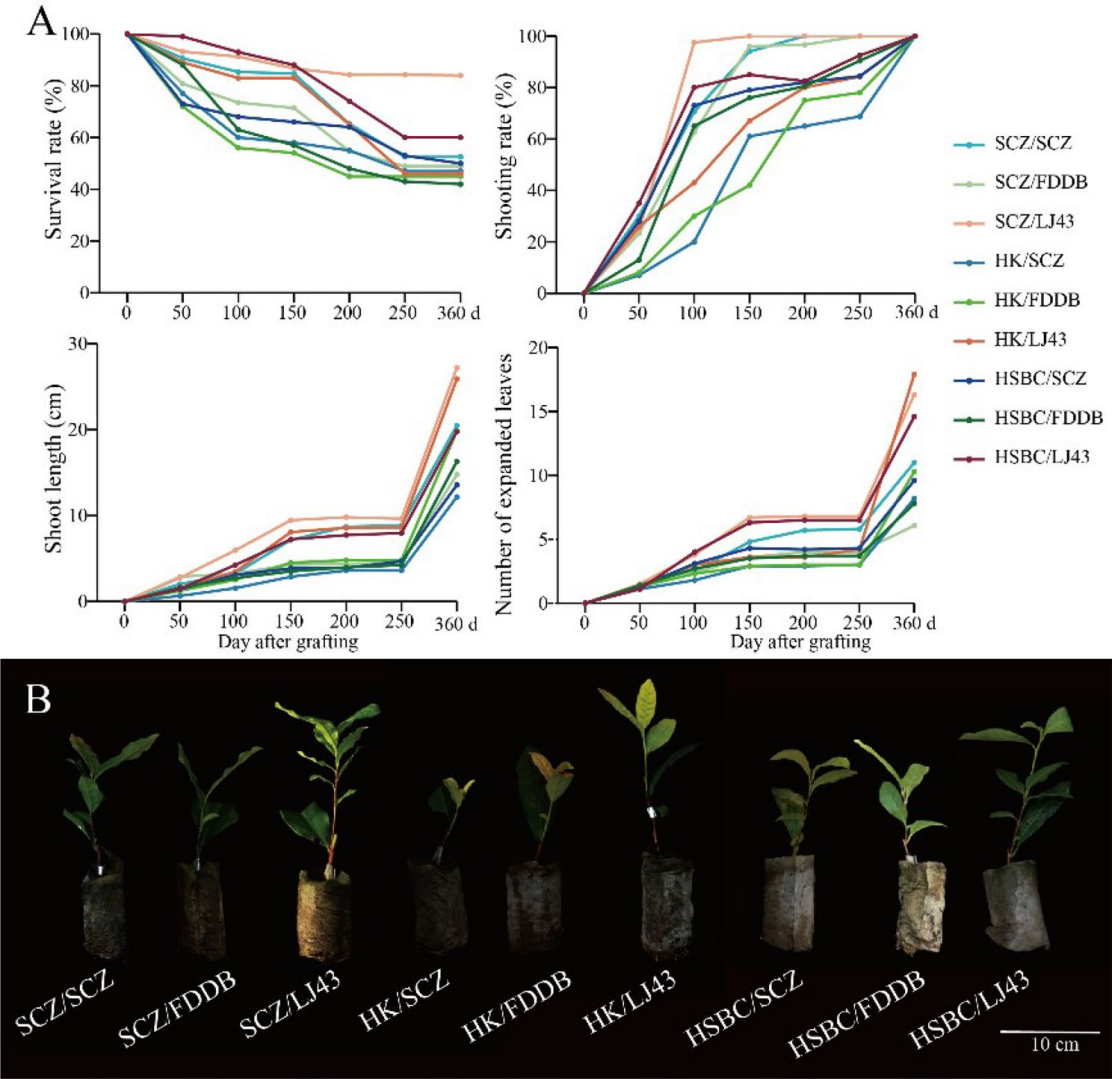
Growth of different tea seedling graft combinations (**A**) and growth performance (**B**) after 360 days of grafting

In recent years, grafting onto appropriate rootstocks has been proven to enhance growth and development of horticultural crop scions [25]. To further compare different tea plant varieties as rootstocks on the compatibility of grafted tea seedlings, the growth status of different tea grafting seedlings was monitored. At 360 days after grafting, the survival rate, shoot length, and number of leaves were higher in LJ43 as rootstocks (Fig. 3A). LJ43 was used as rootstock, and the growth of grafted seedlings was better than that of SCZ and FDDB was observed (Fig. 3B).

**Fig. 4:**
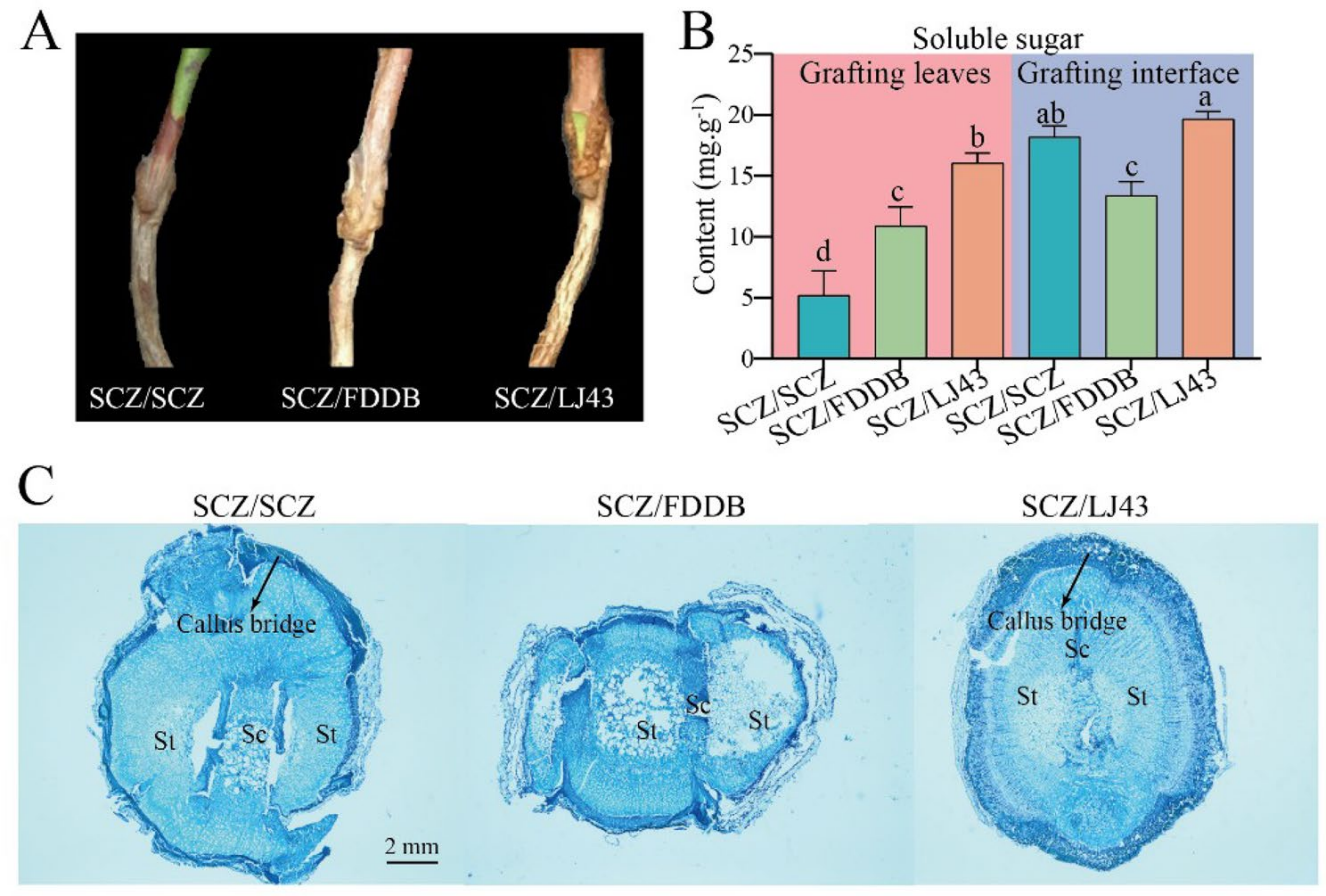
Grafting junctions of different tea seedling graft combinations (**A**) after 360 days of grafting; (**B**) The soluble sugar content of leaves and grafting interface of three graft combinations; (**C**) Anatomical observation on the graft union of grafted tea seedlings with different rootstocks. Scale bars = 2 mm

Graft union formation is a key event in successful grafting during grafting in many plants [26]. Previous study reported that callus proliferation in the vascular connection at the root-scion interface was an important indicator associated with grafting survival [27]. To further determine the compatibility of grafting combinations, sections were made and callus proliferation in the vascular connection at the root-scion interface was observed. With SCZ as the scion as the observation sample, no significant swelling at the grafting interface of the three groups of grafted seedlings (Fig. 4A), and the vascular bundle bridge of LJ43 as the rootstock grafted seedlings was completely established in the later stage (Fig. 4C). Sugar was an activator of important cell division and elongation at the graft junction [28]. To further confirm our above results, the sugar content of the grafting interface and grafted leaves in three grafting seedlings was measured. As shown in Fig. 4B, the soluble sugar content of the SCZ/LJ43 and SCZ/SCZ was significantly higher by 1.47-, and 1.37-fold than those of the SCZ/FDDB in the grafting interface, respectively. However, no significant difference between LJ43 and SCZ rootstocks was found. Consistent with our study, during the healing process of the compatible HZ (“Huaizhi”) grafting combinations, the soluble sugar contents in the junction gradually increased from the initial grafting to the leaf expansion of the scion [29]. In addition, the leaf soluble sugar of SCZ/LJ43 was significantly higher by 3.08-, and 1.47-fold than those of SCZ/SCZ and SCZ/FDDB, respectively. Also, a positive correlation between survival rates and carbohydrate content was found in grafted watermelon leaves [30]. The exogenous application of glucose markedly enhanced callus formation and improved the growth of the cucumber/pumpkin graft [28].

### Effects of grafting on growth indicators and cold tolerance of grafted tea seedlings

**Fig. 5:**
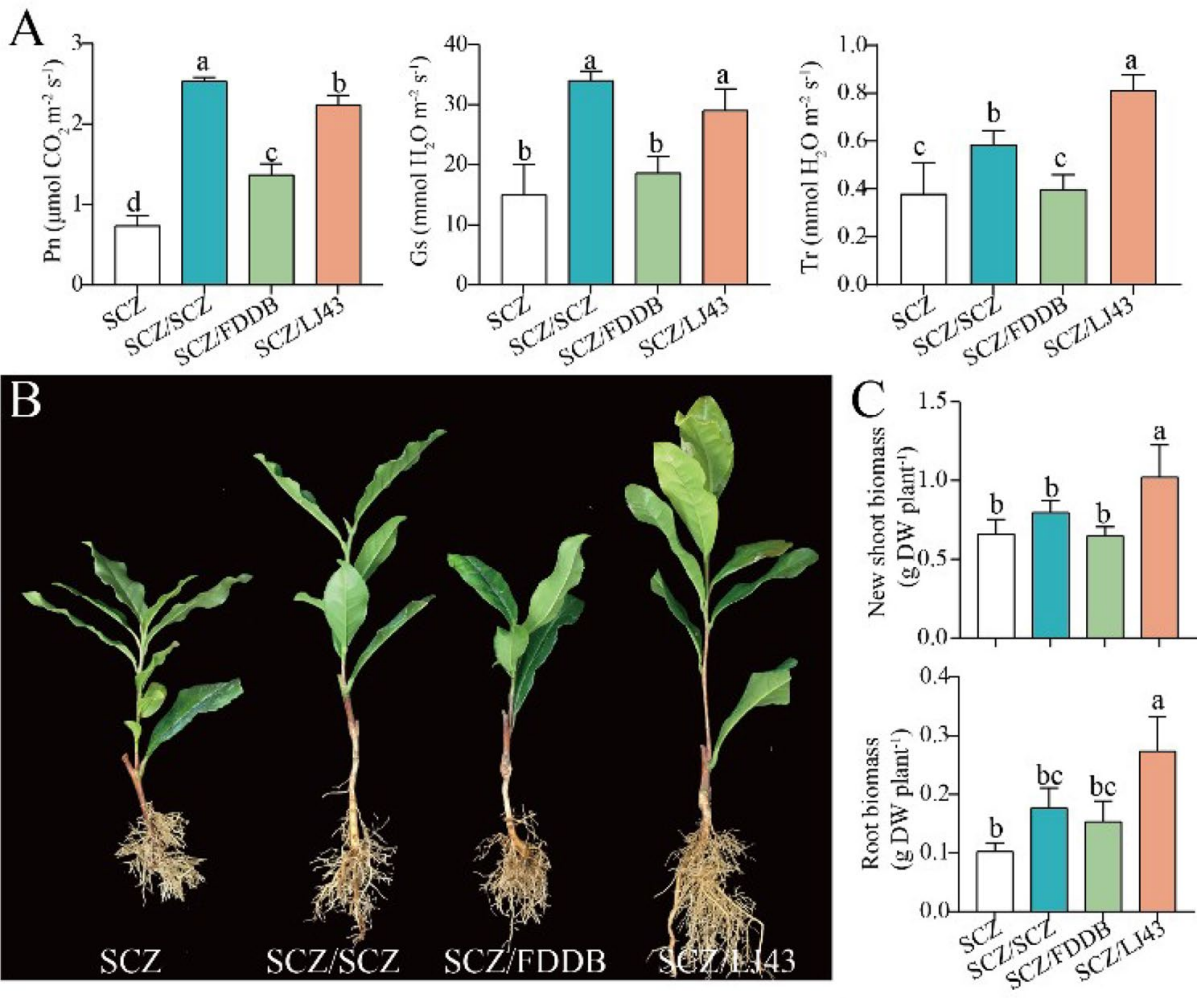
The photosynthetic parameters (**A**), growth performance (**B**) and growth indices (**C**) of three scion/rootstock combinations after grafting using SCZ as scion

To further analyze the growth advantages of grafted seedlings, photosynthesis and growth indicators of grafted and cutting seedlings were monitored. In our study, the Pn (Net photosynthetic rate), Ci (Intercellular CO2 concentration), and Gs (Stomatal conductance) in SCZ/SCZ and SCZ/LJ43 grafted seedlings were significantly increased compared to those of the cutting seedlings (Fig. 5A). Thus, the grafted seedlings of SCZ/LJ43 with a higher level of dry weight in shoots and roots exhibited better growth than the SCZ cutting seedlings (Fig. 5B, C). Previous studies reported that grafting played a critical role in promoting plant growth and photosynthesis performance [31], such as *Carya cathayensis* [32], *Vitis* hybrid ‘Marquette’ [33] and tomato [34].

**Fig. 6:**
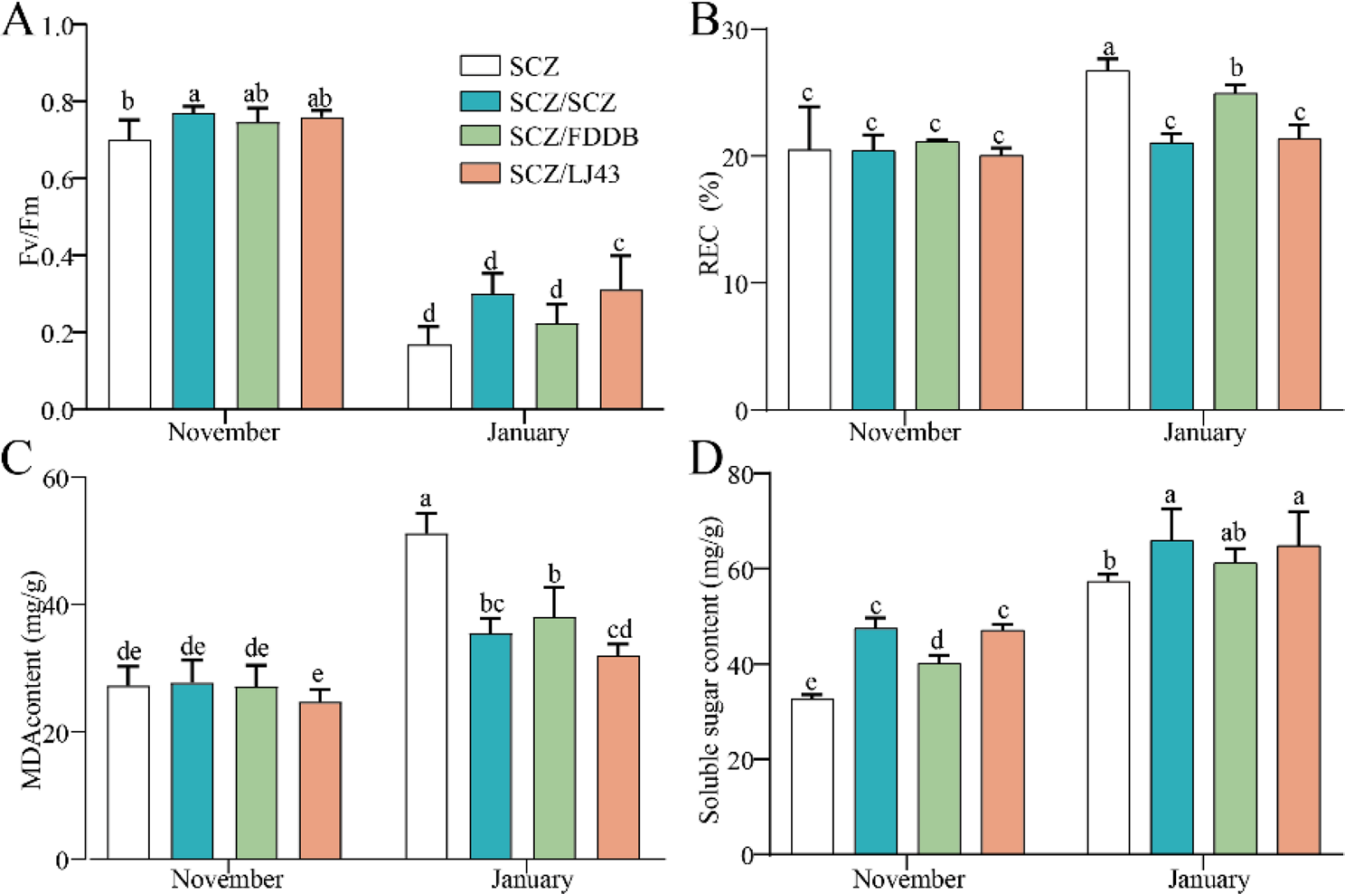
The effect of low temperature on maximum quantum yield of PS II (Fv/Fm), relative electrical conductivity (REC), MAD and soluble sugar content of grafted seedlings of the tea plant

Additionally, grafting also played an important part in plant cold tolerance [35]. It is well known that photosynthesis is an important indicator of response to cold stress in plants [3]. In the present study, the Fv/Fm value of SCZ/LJ43 grafted seedlings was significantly higher than that of cutting seedlings in January, which suggested that grafting enhanced cold tolerance (Fig. 6A). Furthermore, under cold stress, maintaining the integrity of cell membranes is crucial for plant survival [4, 36]. MDA (Malondialdehyde) is the indicator parameter of membrane lipid peroxidation, and an increase in its values suggests an excess of ROS [37]. The efflux or leakage of electrolytes from the cell has been used as another indicator of damage to cell membranes [38]. Therefore, the MDA and electrolyte leakage of leaves were further measured. In our study, the lower levels of REC (Relative electrical conductivity) (Fig. 6B) and MDA (Fig. 6C) in SCZ/SCZ and SCZ/LJ43 grafted seedlings were observed compared with the cutting seedlings in January. In addition, soluble sugar plays an osmoprotective, osmoregulatory, and antioxidant role against ROS accumulation [39]. Compared with the cutting seedlings, the soluble sugar content presented a significant increase in SCZ/SCZ and SCZ/LJ43 (Fig. 6D). It has been reported that grafting onto cold-resistant rootstocks could induce cold tolerance in watermelon [35] and eggplant [40].

Taken together, the results indicated that LJ43 rootstocks reduced membrane lipid damage in leaves of grafted tea seedlings and thereby enhanced the cold tolerance of grafted tea seedlings. However, more work will be done to further illustrate molecular mechanisms of scion-rootstock interaction.

## Conclusion

In our study, the radicle (obtained from the germination of tea seed diameter ≥ 15 mm, D3) as the rootstock for micrografting. The graft combination (GB/R) of green branch (scion) and radicle (rootstock) exhibited a higher survival rate and better growth compared with other grafting combinations (RB/EC, RB/E, RB/R, GB/EC, GB/E). Also, we found that the grafted seedling (SCZ/LJ43) with tea variety SCZ as scion and LJ43 as rootstock showed a higher grafting compatibility, growth, and cold tolerance than those with FDDB (SCZ/FDDB) and SCZ (SCZ/SCZ) as rootstocks. Therefore, our study attempts to establish a feasible tea micrografting method, which may replace tea cutting for tea seedlings to meet the needs of tea cultivation. Even though this study examined the survival rate, growth and cold resistance of micro-grafting without combinations of tea plant varieties, the soil management, light and air humidity still deserve further experimental research on micro-grafting. Future studies can assess scion vigor through more detailed numerical values, and the molecular mechanism of micro-grafting on the cold resistance of tea plants also needs further study.

## Materials and methods

### Plant material

**Fig. 7:**
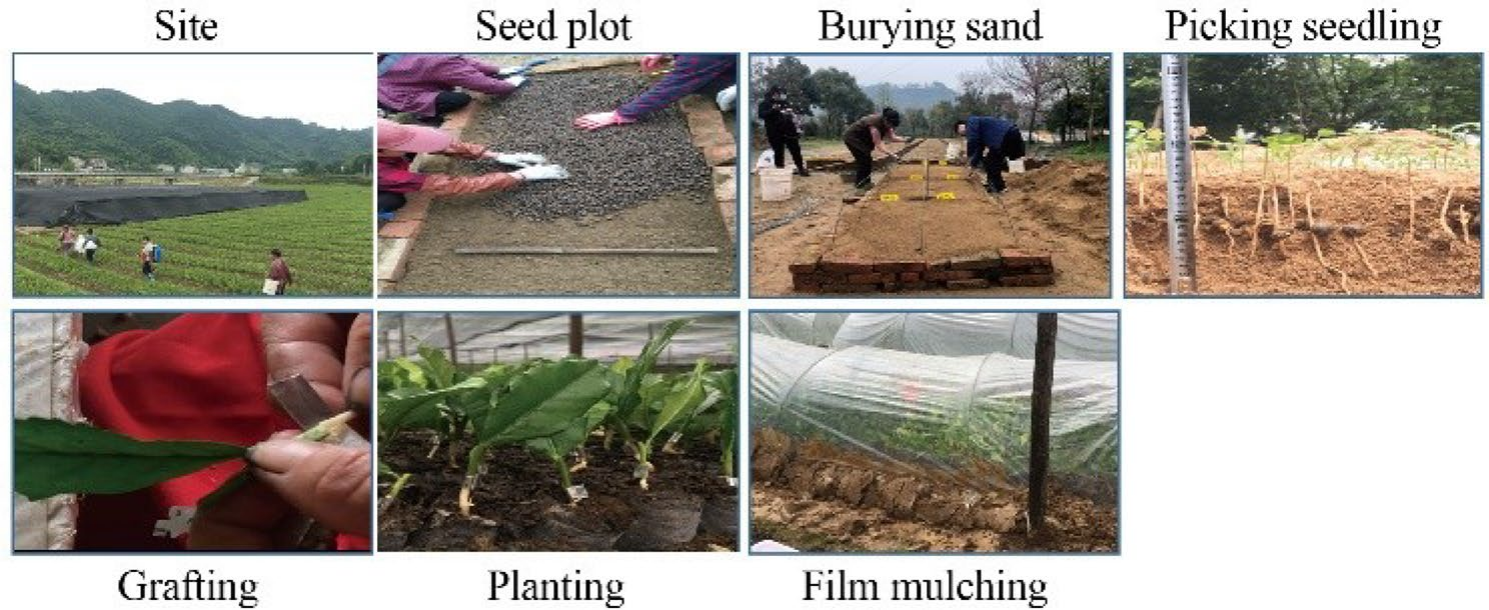
Procedure of grafting on tea plants

This study was carried out at the Dechang Seedling Co., Ltd, located in Shucheng (Luan, Anhui) (116.95′ E, 31.47′ N), which has a subtropical monsoon climate with an average temperature of 16 ℃ to 32 ℃ throughout the year. Tea seeds and scions were provided by Dechang Seedling Co., Ltd, located in Shucheng (Luan, Anhui). Tea seeds were grown in a tunnel-type sand bed built by artificial construction, covered with 16 cm thickness wet sand (Fig. 7). Micrografting (butt-end hypocotyl grafting) was performed with the radicle diameter of the seed was about 2 mm and the green or red branches used as scions. Five different tea varieties ‘Shuchazao’ (SCZ), ‘Fuding Dabai’ (FDDB), ‘Huangkui’ (HK), ‘Huangshanbaicha’ (HSBC) and ‘Longjing 43’ (LJ43) were utilized in our study. The SCZ was selected as scion, and SCZ, FDDB and LJ43 were used as rootstocks to evaluate the tea micrografting compatibility. The growth status of different tea grafting seedlings was monitored from May 20th 2021 to May 20th 2022. During the experimental period, fertilizer and water management practices were the same for all plots. The substrate was uniformly mixed in a uniform ratio (peat: vermiculite: perlite = 2:1:1) during the research study. Covered with moisturizing film, seedlings were cultivated with 85–90% relative humidity. Water-soluble fertilizer containing 0.5% humic acid was applied to the leaves of tea seedlings every month (from June to November 2021).

Subsequently, the grafted seedlings were planted in Shiguan (Yuexi, Anhui) in September 2021, and the cold resistance indicators of the grafted seedlings were measured in December and January (a field experiment).

### Morphological trait measurement

The survival rate was defined as the percentage of surviving branches of the grafted branches in May. Fifteen grafted plants were randomly selected from each graft combination for analysis. The length of new shoots (from the grafting junctions to the top of the canopy) was exactly measured in each treatment.

### Physiological and biochemical indicators measurement

The mature leaves (two leaves) of the new shoots of the scion and grafting junctions were collected after 360 d in May, and the content of total soluble sugar was determined with anthrone colorimetric method (Solarbio, Beijing, China). The samples were exposed to an oven for 1 h at 110 °C, then dry weight (DW) was recorded. The relative conductivity was determined by a conductivity meter (STARTER-3100 C, Ohaus, Shanghai, China). In addition, the net photosynthetic rate (Pn), stomatal conductance to water vapor (Gs) and substomatal CO_2_ concentration (Ci) were measured by a CIRAS-3 Portable Photosynthesis system (PP Inc., USA) [41]. All measurements were conducted from 9:00 to 12:00 am.

### Tissue embedding and microscopic observation

The grafting junctions were harvested for paraffin sections after 360 d in May. The samples were immediately fixed with 70% FAA solution and stored at 4 °C for 48 h after vacuuming. Subsequently, the samples were gradually dehydrated in alcohol and xylene, respectively. The tissue was then placed in a penetrating fluid at 4 °C. Then, a series of operations such as embedding, slicing (10 μm), baking, xylene dewaxing and transparency, toluidine blue solution dyeing, sealing, and baking were conducted. The permanent sections were observed using an optical microscope (LEICA DM500, Wetzlar, Germany).

### Statistical analysis

All the data are the average values of at least three replicates and their standard deviations. Data analyses were conducted through the One-way ANOVA test with SPSS (version 21.0). Duncan’ multiple range test acquired the statistical difference between means.

## Data Availability

The data that support the findings of this study are available from the corresponding author upon reasonable request.
